# Recent Advances in Affinity MOF-Based Sorbents with Sample Preparation Purposes

**DOI:** 10.3390/molecules25184216

**Published:** 2020-09-14

**Authors:** Héctor Martínez Pérez-Cejuela, José Manuel Herrero-Martínez, Ernesto F. Simó-Alfonso

**Affiliations:** Department of Analytical Chemistry, University of Valencia, C/Dr. Moliner, 50, 46100 Burjassot, Valencia, Spain; Hector.Martinez-Perez@uv.es (H.M.P.-C.); jmherrer@uv.es (J.M.H.-M.)

**Keywords:** antibodies, aptamers, molecular imprinted polymers, biomolecules, nucleobases, amino acids, carbohydrates, sample treatment, solid-phase extraction, metal–organic frameworks

## Abstract

This review summarizes the recent advances concerning metal–organic frameworks (MOFs) modified with several biomolecules (e.g., amino acids, nucleobases, proteins, antibodies, aptamers, etc.) as ligands to prepare affinity-based sorbents for application in the sample preparation field. The preparation and incorporation strategies of these MOF-based affinity materials were described. Additionally, the different types of ligands that can be employed for the synthesis of these biocomposites and their application as sorbents for the selective extraction of molecules and clean-up of complex real samples is reported. The most important features of the developed biocomposites will be discussed throughout the text in different sections, and several examples will be also commented on in detail.

## 1. Introduction

Metal–organic frameworks (MOFs) are a hybrid porous network built by the coordination of metal ions/clusters with organic ligands introduced for the first time by Yaghi’s group [1]. The resulting crystalline structure shows a two- or three-dimensional skeleton with unique properties [2]. Regarding the MOF catalog, most metal ions can be used to synthesize them and, accordingly, the reported number of these materials is enormous. Indeed, the accurate selection of precursors can lead to building blocks with different compositions, topologies, etc. Both metal ions and organic linkers play key roles in the final structure of the materials and their properties. MOF characteristics that can be highlighted are their high surface areas, tunable chemistry, and uniform structured nanoscale cavities [3]. In addition, the tailorable surface of these materials through several synthetic strategies (pre-, post-, etc.) [4] allows the introduction of desirable functionalities for concrete requests. Furthermore, MOFs possess flexible frameworks different to traditional rigid porous inorganic materials such as zeolites and porous silica [5]. All these good features have caused MOFs to be attractive materials for several applications covering extensive areas, such as catalysis [6], gas separation [7], pollutant removal [8], and the biomedical field [9], among others.

In recent years in the analytical chemistry field, researchers have been engaged in the development of novel materials and strategies to improve analytical methodologies in terms of efficiency, selectivity, cost-effectiveness, and detection limits to face recent analytical challenges. In fact, the MOFs in sample treatment have been directly employed or designed to meet the demands of analytical applications [10]. These materials can be used in different formats (bulk powder, fiber coating, membranes, etc.) [11], and they are convenient for sampling, preconcentration, or the clean-up of complex matrices due to their abovementioned features. The analytical performances obtained with MOFs in the sample treatment field include adsorption efficiency, good reproducibility, satisfactory reusability, convenient shape- and size-selectivity, and large preconcentration factors. However, despite their good characteristics, the direct use of conventional MOFs as micro-/nanocrystals is somewhat restricted, since they have limited chemical selectivity, which is largely conditioned by the ligand. Additionally, the need for selecting judiciously the organic linker is a major concern, since it affects directly the analytical performance of the resulting framework [12]. Thus, there are many organic compounds that can be used as linkers (e.g., amine-, ciano-, and carboxyl ligands, etc.), although the use of cationic/neutral ligands is scarce due to their low affinity towards metal cations [13,14]. Additionally, most of the traditional ligands adopted present a low grade of functionality and a reduced biocompatibility, thus limiting their use as (bio)-materials in sample treatment, point-of-care diagnostics, and bio-carriers, among other applications. Consequently, the requirement for a new generation of MOFs with all these abovementioned aspects is mandatory.

In the recent years, biological metal-organic frameworks (bio-MOFs) have fascinated researchers in the analytical field, not only for their sustainable aspects, but also for their superior features versus common organic ligands (2-methylimidazole, trimesic acid, etc.). These materials are made up of biologically related ligands (bio-ligands: amino acids, nucleobases, proteins, etc.) and metal ions [15]. The superior performance of biomolecules as building blocks to form bio-MOFs can be summarized as follows: multiple possible coordination modes, self-assembly properties, biocompatibility, economic feasibility, flexibility, inherently chirality, and multiple functional groups [14]. In this sense, it is not difficult to think that the application of these bio-MOFs in several fields (enantioseparation, selective capture, etc.) have aroused, although other well-known MOFs have been applied since their discovery [14,15].

**Figure 1:**
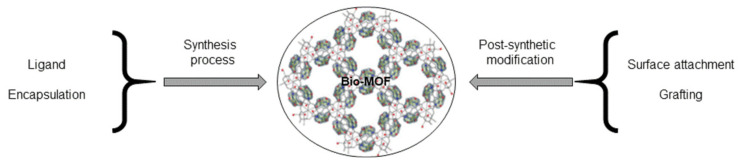
Synthetic routes for the development of biocomposites based on the incorporation of biomolecules in MOFs.

As commented on above, due to the diversity of the available building blocks and the ease of the modification and functionalization of MOF materials, these frameworks can be effective supporting structures for hosting biomolecules. In this sense, pre- and post-functionalization strategies [6,16,17] have been adopted for this concern (see Figure 1). Within the pre-functionalization approaches, there are two choices: (i) using the biomolecule (e.g., amino acids, nucleobases) as ligands during the synthesis bio-MOF procedure and (ii) the direct encapsulation of the biomolecule (e.g., proteins) within the framework. Alternatively, a post-synthetic modification strategy can be applied, where the surface attachment of the biomolecule (e.g., ATP) or grafting the biomolecule (e.g., aptamers/antibodies) in the MOF host can be accomplished. Thus, the introduction of biomolecules as ligands during the synthesis procedure could be considered as the simplest strategy, although the certain interaction ability of bio-MOF is reduced, since some biomolecules are trapped in the framework. Regarding the post-synthetic functionalization approach, these protocols are more time-consuming, although they improve the ability of the incorporated biomolecules to interact with analytes.

The present review summarizes the most recent advances (the last 5 years) focused on the incorporation of different biomolecules (amino acids, carbohydrates, etc.) as well other selective structures (antibodies, aptamers, and molecular imprinted polymers (MIPs)) in MOF supports to prepare affinity-based sorbents (bio-MOFs) for extraction, preconcentration, or clean-up purposes. Different applications of the above-mentioned materials will be discussed throughout the text, and the possible challenges will be also considered.

## 2. Applications of Affinity-Based MOF Materials in Sample Treatment

Despite the sophisticated and highly sensitive techniques that have been developing in the analytical field in the last few decades, the determination of analytes in complex samples such as environmental, food, or biological matrices requires previous and efficient pretreatment steps in order to make analytes suitable for their introduction in the analytical system. Within sample preparation techniques, solid-phase extraction (SPE) is the most common technique used for isolation and enrichment purposes [16,17,18,19,20]. In this sense, the abovementioned bio-MOFs and their features [21] can be a good alternative to commercial SPE sorbents in order to provide high extraction efficiencies, a good clean-up in short analysis times, and a low cost. In any case, the success of any affinity-based sorbent is strongly influenced by the ligand agent choice, as this will be incorporated in the MOF support. For this purpose, available ligands obtained from biological sources (amino acids, proteins, carbohydrates) with certain selectivity can be used to produce the so-called bioaffinity-based MOFs. On the other hand, highly specific ligands such as antibodies or other biomimetic recognition elements (such aptamers and MIPs) can be employed in combination with MOFs for the selective molecular recognition of target analytes. Taking into account this distinction, the most recent contributions will be grouped and discussed in terms of the type of bioligand.

### 2.1. Bioaffinity-Based MOFs

Amino acids can be described as organic compounds with carboxylic (-COOH) and amine (-NH_2_) functional groups in their structure, along with the R group for individual amino acids. These groups and organic chain dominate their physical and functional properties. Their rich coordination chemistry and intrinsic chirality make these compounds suitable ligands for the preparation of bio-MOF structures. The most common technique to incorporate amino acids in bio-material is by adding these biomolecules to the precursor mixture [22]. The resulting bio-MOFs have been applied in several fields, such as chiral recognition [23,24], the recovery of heavy metals [25,26], or the enrichment of peptides [27,28]. Table 1 shows an overview of the most important and recent applications using amino acids as ligands to prepare bio-MOF supports as SPE materials. Concerning the retention mechanism, the presence of amino and carboxylic groups in these ligands can favor electrostatic and hydrogen-bond interactions with the analytes. Apart from these interactions, some of these systems offer a controllable ordered porosity, which can be used to tune the guest-to-framework interaction, as will be commented on below.

In particular, Mon and co-workers [26] have reported the design and synthesis of several three-dimensional frameworks using different amino acids such as L-serine, L-methionine, and L-threonine. The best sorbent for mercury removal was that built with L-methionine, with the formula {Cu_4_(II)[(S,S)-methox]_2_}·5H_2_O, where methox is bis[(S)-methionine]oxalyl diamide. This is due to its narrow functional channels decorated with thioalkyl chains, which were able to capture HgCl_2_ from aqueous media in an efficient and selective way. Indeed, this bio-MOF was applied in dispersive -SPE mode, being able to reduce the dangerous level of 10 μg mL^−1^ of Hg(II) to acceptable limits (<2 μg L^−1^) in drinking water with an extraction efficiency close to 100% in a short time (15 min).

In a recent study, the same authors in collaboration with our research group [29] have developed a bio-MOF derived from the amino acid L-serine, which was evaluated as an SPE sorbent for the molecular recognition and extraction of B-vitamins. The determination of the target analytes was carried out by HPLC-UV. The functional pores of this bio-MOF exhibited high amounts of hydroxyl groups, jointly directing other supramolecular host–guest interactions and thus providing the recognition of B vitamins. The sorbent showed an acceptable reusability (five times) and required fewer amounts (25 mg) than other commercial phases (150 mg–10 g). Other quality analytical parameters of the method included quantitative extraction recoveries for energy drink and juice samples (75–123%), an acceptable reproducibility (RSD values < 14%), and low limits of detection (LODs, 0.4 to 1.4 ng mL^−1^).

Other interesting applications of these bio-sorbents were reported by Wu et al. [30], who developed a magnetic multifunctional MOF, denoted Fe_3_O_4_@PDA@MIL-125@Au@L-Cys. Thus, magnetite nanoparticles (NPs) were synthesized and coated with poly(dopamine) (PDA), which acted as linked layer to construct MIL-125 by self-assembly. After that, AuNPs were attached to the surface of MOFs, followed by the incorporation of L-cysteine (L-Cys). The composite material was applied to selectively extract glycol- and phosphopeptides from the tryptic digests of standard proteins (horseradish peroxidase and β-casein). The sorbent exhibited an excellent sensitivity (0.1 fmol μL^−1^) and satisfactory reusability (five cycles). The retention mechanism of the sorbent indicated a typical hydrophilic interaction chromatography (HILIC) mechanism where both the adsorption and partitioning of these polar solutes contributed. The proposed system was applied in the human crystalline lens, where 81 N-linked glycopeptides corresponding to 35 glycoproteins and 175 phosphopeptides ascribed to 55 phosphorylated proteins could be identified, respectively.

Before discussing the preparation of bio-MOFs with proteins, peptide@MOF composites will be commented on, since this might give us an insight into how more complex molecules (proteins) can fold or “move” in these hybridizations [34,35]. Each peptide has a different sequence of amino acids, which influences its conformation and stereochemical configuration [36,37]. Their coordination with metal ions is similar to amino acids based on O or/and N atoms [36,38]. The intrinsic chirality of these biomolecules makes them good candidates to produce functional chiral MOFs. Despite these good features, bio-MOFs containing peptides in their structure have been scarcely explored in sample treatment [12,39,40]. The expected recognition mechanism of these composites should be similar to that those derived from amino acids. Additionally, peptides as linkers can promote host–guest interactions due to their ordered structures and adaptable porosity.

In a recent study, Stylianou and collaborators [12] have described the use of a chiral peptide, glycyl-L (S)-glutamate, to construct chiral and rigid ladder-type building units that were subsequently used in an isoreticular homochiral peptide-based MOF. The pore size of this family of homochiral MOFs was tuned through judicious choice of the type and size of linker. The resulting peptide-based MOFs were tested for their enantioselective adsorption of chiral molecules (glycidol and hydrobenzoin), and it was confirmed that their separation is size-dependent.

**Figure 2:**
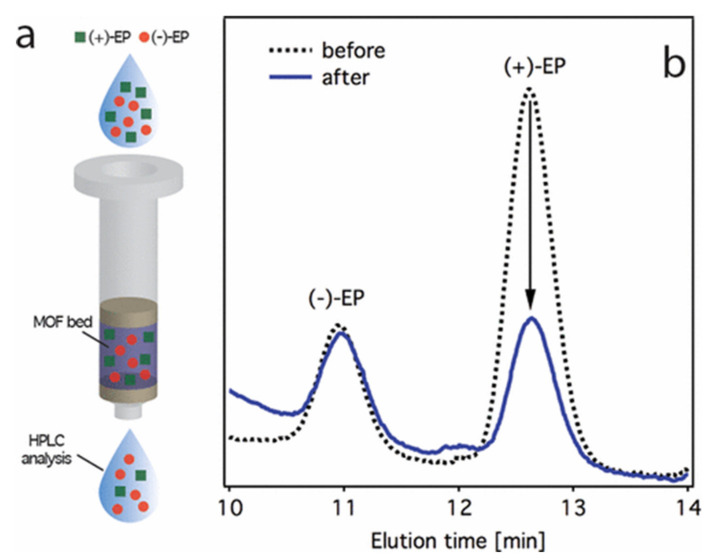
(**a**) SPE separation of EP in hexane:EtOH 75:25 using Cu(tripeptide Gly-L-His-Gly) as a chiral bed. (**b**) HPLC chromatograms of ephedrine racemate before (dashed line) and after (solid line) passing through the MOF bed. Reprinted with permission from [40]. Copyright 2017 American Chemical Society.

In another work, Navarro and his coworkers [40] developed a bio-MOF based on tripeptide Gly-L-His-Gly for the purification of (+)-ephedrine (EP) enantiomer. This bio-MOF was used as an SPE phase, being able to isolate (+)-ephedrine enantiomer from a racemic mixture in 4 min. The determination of the target analytes was carried out by HPLC-fluorescence detection (FLD). Figure 2 shows the chromatograms of the sample before and after the SPE treatment with bio-MOF, thus confirming the ability of this material to preferentially trap (+)-epinephrine. The authors demonstrated that the chiral recognition is due to the preferential binding of one of the enantiomers because of additional H-bonds with the framework that led to more stable adducts.

Other application of peptide-based MOFs is the extraction of glycopeptides in the field of glycoproteomics. For instance, Liu et al. [39] developed a glutathione-functionalized magnetic MOF for the efficient enrichment of N-linked glycopeptides. For this purpose, an amino-derived MOF (UiO-66-NH_2_) was coated on the surface of magnetic Fe_3_O_4_ with hydrophilic PDA as a linker, following the immobilization of AuNPs on the MOF. Finally, glutathione was linked to the MOF via the affinity between Au and the thiol group. The as-prepared magnetic bio-MOF exhibited a high selectivity (1:100) and great sensitivity (0.5 fmol μL^−1^) towards glycopeptides. Furthermore, the developed sorbent was able to identify 273 glycopeptides, corresponding to 94 glycoproteins in only 2 μL of human serum.

Concerning protein–MOF systems, few studies have been reported with analytical treatment finalities due to the large and delicate conformational structure of proteins and their low stability in harsh conditions. The bulky structures of proteins can cause some problems in incorporating them on the surface or the skeleton of MOFs. Thus, adjustments of MOF pore/channel engineering is required to accommodate these large biomolecules [41,42]. A few works have reported protein immobilization in zeolitic imidazolate framework (ZIF) structures [42,43,44]. Chen and co-workers [42] described a versatile biomimetic strategy for the high-efficiency encapsulation of biomacromolecules within MOFs. This strategy relied on the formation of Cys-triggered prenucleation clusters around proteins, followed by the production of MOFs around the proteins. Twelve proteins, including enzymes, with different surface chemistries were successfully encapsulated into ZIF-8 within several minutes. The encapsulated proteins maintain their native conformations. The proposed method gave a higher encapsulation efficiency (up to 18.2 wt.%) than most of the reported MOF-encapsulated proteins formed. In another work, Liu [44] fabricated hierarchical micro/meso-ZIF-8 using cetyltrimethylammonium bromide as a structure-controlling agent and L-histidine as co-template. The as-prepared MOF contained proper micropores and maximum mesopores (of ca. 35.6 nm) that favour the adsorption of lysozyme. The sorbent showed a high adsorption capacity (377 mg of protein per gram of sorbent) and an acceptable reusability (at least four times without significant losses). Both the works cited herein open a broad application outlook of hierarchical protein@MOFs in molecule separation, immobilization, and enrichment. The non-destructive encapsulation, the speed of the process, and the controlled release of the encapsulated biomolecules will be the challenge in the coming future for several (bio)applications.

With regards to enzyme-MOF composites, the hybrid nature of MOFs plays an important role in enzyme stabilization. One of the key aspects in the enzyme immobilization is the compatibility of the bio-system to avoid the protein denaturation and undesired leaching during its use. As indicated above, MOFs represent valuable platforms to immobilize enzymes and minimize undesirable losses of these in complex matrices, thus improving the biocomposite stability [45]. Feng’s group [46] described a strategy to capture enzymes in the pore structure of PCN-133 (Al). This MOF contains mesocages (5.5 nm) that can act as single-molecule traps for the encapsulation of a single-enzyme molecule. The biocomposite showed satisfactory loading capacities (ranged between 890 and 1000 mg g^−1^ of sorbent depending of the enzyme), being higher than those achieved using mesoporous silica sorbent (SBA-15). Furthermore, the immobilized enzymes showed almost no leaching during recycling and kept a high catalytic activity.

**Figure 3:**
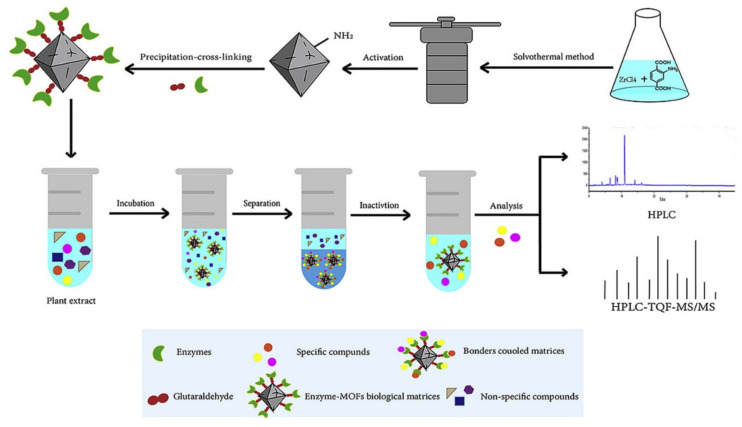
Schematic representation of Bio-MOF (UiO-66-NH_2_@enzyme) synthesis and extraction procedure of bioactive components from a plant extract. Reprinted from [47] with permission from Elsevier. Copyright 2019.

Recently, the immobilization of enzymes onto MOFs has been used as a promising support for “ligand fishing” to obtain bioactive compounds directly from complex mixtures. In particular, Chen and co-workers [47] have reported the use of MOF UiO-66-NH_2_ as a host to immobilize porcine pancreatic lipase (PPL) via the precipitation cross-linking method. The preparation protocol of this material and the main steps of the SPE procedure are shown in Figure 3. After incubation of an extract of a plant (*Prunella vulgaris L.*) with the biocomposite, several bioactive components were obtained and identified by HPLC-Q-TOF-MS/MS. The PPL@MOF provided the merits of a larger protein loading capacity, higher activity recovery, and convenience of preparation. Due to the merits of the proposed strategy, it is expected that it can be applied to a wide range of target proteins to screen bioactive components from complex mixtures, which undoubtedly would accelerate the discovery of new drug candidates.

**Figure 4:**
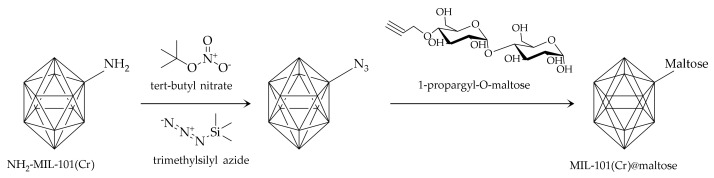
Schematic synthesis route for MIL-101(Cr)@maltose. Adapted from [49] with permission from RSC, 2018.

Carbohydrates, from simple molecules (e.g., mono- or disaccharides, such as maltose) to complex structures (e.g., polysaccharides, cyclic oligosaccharides as cyclodextrins (CDs)), are a highly desired class of bioligands for MOF synthesis, since they are composed of edible components; thus, their biocompatibility and environmentally friendly nature are strongly highlighted [48]. Concerning the immobilization of small carbohydrate ligands, maltose has been commonly used. The resulting materials with surface-exposed maltose groups showed a strong hydrophilic interaction with polar analytes (e.g., glycopeptides). Thus, Ma and colleagues [49] developed a MIL-101 (Cr)-maltose material for the selective enrichment of N-linked glycopeptides. The schematic synthetic route is shown in Figure 4. The significant features of the method were a low LOD (1 fmol) and large adsorption capacity (150 mg g^−1^). Additionally, after enrichment with the developed sorbent, up to 33 glycopeptides from the tryptic digest of human IgG could be observed with high intensities.

In another work, Giménez et al. [50] developed a biocomposite obtained by the post-synthetic modification of ZIF-8 MOF with a maltose-exposing biocompatible surfactant (n-dodecyl β-d-maltoside) to increase the affinity of the hybrid material toward concanavalin A (lectin protein). The resulting material with surface-exposed sugar moieties presented an affinity toward lectin protein concanavalin A.

Additionally, complex polysaccharides such as chitin have been used to synthesize biocomposites. In particular, Wisser and coworkers [51] used chitin as a support for Cu_3_(BTC)_2_ MOF deposition. The resulting biocomposite was used as an adsorption material for toxic industrial chemicals, such as ammonia (39.3 mg g^−1^). This feature indicates that this composite shows a high potential for filtration applications for toxic industrial gases—for example, in air filters.

Cyclodextrins (CDs) from natural products display the –OCCO– binding motif on both their primary and secondary faces, auguring well for forming extended structures with Group IA and IIA metal and host–guest complexes. Apart from these features, their water solubility, biocompatibility, and non-toxicity make them an interesting option to combine with MOFs in a variety of applications, where traditional MOFs face limitations [52]. Besides, the cavity of CDs can allow the penetration of nonpolar solutes of proper size and their binding to the CDs through host–guest interactions.

The first example of CD-MOF for sample treatment purposes was proposed by Liu et al. in 2017 [53]. The authors developed a magnetic copper-based MOF containing β-CD for the adsorptive removal of neonicotinoid pesticides. The β-CD-MOF was prepared using Fe_3_O_4_–GO–β-CD as a starting material. The adsorption capacity of the biocomposite for tested insecticides ranged from 1.77 to 3.11 mg/g. The efficiency of insecticide removal by β-CD-MOF was tested using tap water samples (for 1 h), obtaining that all the neonicotinoid insecticides in these samples were removed at concentrations below 0.5 μg mL^−1^. These results showed that the hydrophobic inner cavities and supramolecular recognition of Fe_3_O_4_–GO–β-CD significantly improved the adsorption capacity and rate of composite for neonicotinoid insecticides.

In another work, Li et al. [54] developed a CD-MOF assembled with γ-CD and K(I) used as an SPE phase for the preconcentration of sulfonamides from meat samples prior to HPLC-UV determination. The CD-MOF exhibited a high adsorption capacity and a quick kinetic equilibrium adsorption of sulfonamides. Under optimized conditions, the developed method showed high recoveries (76–102%) in food samples (chicken, pork, liver, and fish), satisfactory precision values (RSD < 6.5%), and low LODs (0.32–1.7 μg L^−1^).

### 2.2. Immuno- and Mimetic-Based MOFs

Up to here, the selectivity or recognition ability of most bio-MOFs described in the previous section have been attributed to hydrophobic, electrostatic, and π-interactions of solutes with the functional ligands (commonly aromatic), although the selectivity of some of these can be enhanced by tailoring the size and shape of the bio-MOF pore to match with the guest. In order to enhance the selectivity of the sorbents via molecular recognition, much effort has been made in the last decade to develop affinity sorbents such as antibodies, aptamers, or MIPs [55,56]. The need to improve the selectivity of MOFs has increased research on the use of antibodies or other biomimetic recognition elements such aptamers or MIPs immobilized on MOF supports. In this section, the most relevant contributions in the sample treatment field will be briefly discussed, and an overview of the applications is summarized in Table 2.

Antibodies combined with MOFs have been mainly used as immunosensing platforms in several applications, such as the recognition of tumor biomarkers in biological fluids [57] or the detection of pollutants in environmental and food samples [58]. Despite these interesting applications, the use of these biocomposites as sorbents in the extraction and enrichment of analytes during sample preparation has been scarcely reported [59,60]. Indeed, these works have been focused on the isolation of pathogenic bacteria. In particular, Yim et al. [59] immobilized antibodies on ZIF-8/Fe_3_O_4_ hybrid NPs for capturing *Staphylococcus Aureus* in several dairy products. Half fragments of monoclonal Staphylococcus antibodies were conjugated to low-coordinated Zn sites located on the outer layer of ZIF-8 via Zn–S bonding. After the capture and magnetic separation of *Staphylococcus* in milk using these hybrid NPs, the bacteria concentration was determined with a portable luminometer, reaching an LOD of 300 cfu mL^−1^.

Aptamers are RNA or DNA single-stranded oligonucleotides (20–100 base pairs) that show a high affinity toward the target molecule(s). Regarding their synthesis process, they are built by an in vitro combinatorial selection method called the systematic evolution of ligands by exponential enrichment (SELEX), an iterative process where aptamers that specifically bind to the proposed target are selected and properly expanded [61]. Aptamers are highly comparable to antibodies, although they show several advantages, such as cheap chemical fabrication, superior stability, and high reproducibility. Despite these unique properties, these molecules have been scarcely incorporated in MOF supports for sample treatment purposes due to their relatively short existence time [62,63,64,65,66]. Lin and co-workers [62,63] developed two aptamer-MOF devices for the selective extraction of polychlorinated biphenyls (PCBs) in soil and fish samples followed by GC-MS analysis. In their first work [62], the MOF (in particular, MOF-5) was first synthesized via electrodeposition in a stir bar, which was used as a platform for the aptamer immobilization. This aptamer-functionalized stir bar sorptive extraction (Apt-MOF SBSE) fiber was facile synthesized in one step. The Apt-MOF SBSE pretreatment coupled with GC–MS exhibited a large selectivity, satisfactory recovery (89.2–97.1%), good enrichment factor (1930–2304), low LOD (0.003–0.004 ng mL^−1^), and reproducibility (fiber-to-fiber RSD < 5.0%) for the detection of PCBs. In the second work [63], the authors synthesized an aptamer-functionalized magnetic MOF material (Fe_3_O_4_@PDA@UiO-66-Apt) as a sorbent for dispersive SPE for the selective enrichment of PCBs from soil samples. First, the synthesis of magnetic amino-functionalized UiO-66 (Fe3O4@PDA@UiO-66-NH2) using PDA as a covalent linker was performed. Then, amino-functionalized aptamers were able to recognize 2,3′,5,5′-tetrachlorobiphenyl (PCB72); 2′,3′,4′,5,5′-pentachlorobiphenyl (PCB106) was covalently immobilized on UiO-66-NH_2_ through the coupling reagent of glutaraldehyde. The merits studied in this study included the high selectivity and reusability (up to 60 cycles with recoveries over 80%) of the sorbent, satisfactory recoveries (89.2% to 95.2%), and low LODs (10–15 ng L^−1^).

Jiang et al. [64] prepared an aptamer-functionalized magnetic conjugated organic framework (COF) for the selective extraction of traces of PCBs in human serum. For this purpose, a COF rich in carboxyl groups was first synthesized on the surfaces of magnetic cores, followed the immobilization of aptamer (bearing amino groups) on the surface of the magnetic COF. The resulting Fe_3_O_4_@COF-Apt showed a good reusability (at least 10 extraction cycles with recoveries of >90%), recoveries in the range of 87.7–101.5%, and a very low LOD (2.1 ng L^−1^).

Additionally, aptamer-functionalized MOF materials have been applied for food safety purposes [65,66]. As an example, Zhang et al. [65] synthesized a magnetic MOF (MIL-101) coupled with an aptamer for the enrichment of ochratoxin A in corn and peanut samples, and the determination was followed by UHPLC-MS-MS. The analytical features of the optimized method were as follows: a satisfactory recovery (82.8–108%), good loading capacity (553 pmol mg^−1^), acceptable reusability of sorbent (at least 12 times), and low LOD (67 pg L^−1^).

**Figure 5:**
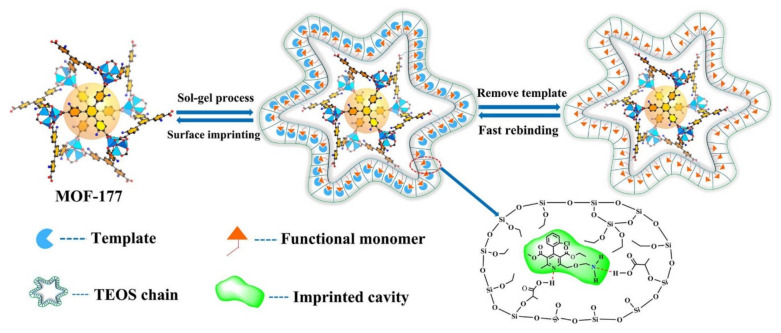
Process of the sol-process synthesis of MOF-177@MIP and the resulting imprinted cavities in the hybrid material. Reprinted from [67] with permission from Elsevier. Copyright 2019.

As we mentioned above, increasing attention has been given to the development and application of MIPs as synthetic receptors as promising alternatives to biological receptors, since they offer a more stable, robust, and cheap alternative for molecular recognition [56]. Molecular imprinting implicates the use of functional monomers, cross-linking agents, and template molecules to create selective artificial cavities in a three-dimensional polymer network. The polymerization is carried out in the presence of template molecules (represented by the target analyte), following its posterior removal from the polymer matrix. The resulting cavities are complementary in size and shape with the target analyte, thus providing interaction sites to recognize the target or structurally related compounds [56]. Taking advantage of the properties of MIPs, their hybridization with MOFs represents a new, emerging research field to explore. In the last two years, several examples of these novel materials have been reported [67,68,69,70,71]. For instance, Wei and coworkers [67] fabricated a core-shell hybrid MIP based on MOF (MOF-177) to selectively determine S-amlodipine (used as an antihypertensive and antianginal agent) in water samples (Figure 5). In the polymerization system, the drug was used as a template, methacrylic acid as a functional monomer, tetraethoxysilane as a crosslinker, MOF-177 as a core support material, and acetic acid as a catalyst. After optimizing the parameters of polymerization, the sorbent showed a large adsorption capacity (1.31 mmol g^−1^) and imprinting factor (5.79), being superior to other preparation methods of sol-gel hybrid MIPs reported in the literature.

Additionally, MOF@MIP materials have been also applied to drug and food analysis [68,69]. For example, Wang et al. [68] developed an MIP material based on NH_2_-MIL-101(Cr) as a sorbent in dispersive micro-SPE to extract diclofenac in urine samples. Under optimum conditions, the average recovery ranged from 88.3% to 101.6%.

Another example of mimetic-MOF based sorbents was developed by Liang and collaborators [69]. In this case, a surface-imprinted polymer was prepared using UiO-66-NH_2_ as a MOF support, and the final affinity sorbent was applied to the enrichment of trace aflatoxins (B1, B2, G1, and G2) in grain samples. For this purpose, UiO-66-NH_2_ previously modified with glycidyl methacrylate was mixed with the dummy template (quercetin) in a solution containing acrylamide, ethylene glycol dimethacrylate as a crosslinker, and azobisisobutyronitrile as an initiator of the subsequent polymerization reaction. Under optimized conditions, the prepared SPE cartridges containing the UiO-66-NH_2_@MIP gave adsorption capacities from 4 to 10 mg g^−1^, extraction efficiencies of 74–99%, and LODs that ranged between 90 and 130 ng kg^−1^. The performance of method was compared with different commercial SPE cartridges (immunoaffinity columns, C18, Florisil, and silica-based), showing better selectivity, adsorption properties, and cost than the commercial sorbents.

### 2.3. Miscellaneous Affinity Ligand-Based MOFs

In addition to the ligands mentioned in the preceding sections, many other biomolecules (such as nucleobases, nontoxic carboxylic acids, and biopolymers, among others) have been also used as affinity media to develop efficient and selective extraction sorbents (see Table 3 for selected examples).

Over the past few years, nucleobases have been widely employed for the synthesis of bio-MOFs. These biomolecules have many oxygen and nitrogen donor groups and aromatic rings that can form several interactions (for example, by coordination and hydrogen bonding) with metals. Within the nucleobase-derived bio-MOFs, the most common are those based on adenine (Ade) [72]. This is due to the following issues: i) adenine offers five metal binding sites (N1, N3 from pyrimidinate, N6 from the amino group, N7, and N9 from imidazolate) for interaction/coordination; ii) its rigid structure allows the formation of a permanent and high porosity; iii) affinity with several metal ions; and iv) ability to adsorb small molecules, such as carbon dioxide [14]. Indeed, several studies have been reported of MOFs with nucleobases as sorbents for gas adsorption [72], and applications focused on extracting compounds from environmental samples [73,74,75,76].

Liu and co-workers [74] fabricated bio-MOF-coated SPME fibers for the extraction of PAHs and organochlorine pesticides in environmental water pesticides samples followed by GC-flame ionization detector (FID) determination. For the fabrication of bio-MOF-coated fibers, a neutral silicone sealant was used as the bridging agent with the help of the solidification reaction. A series of isoreticular bio-MOFs 100, 101, and 102 with permanent mesoporosity and different pore sizes and surface areas (3222–4410 m^2^ g^−1^) were synthesized by the solvothermal method and stepwise ligand exchange reaction. The analytical performance of the bio-MOF-102 fiber, which gave the best extraction ability, due to a pore structure--dominated mechanism was also evaluated. The method showed satisfactory extraction recoveries (80–111%), low LODs (0.22–2.3 ng L^−1^), and an acceptable reproducibility (RSD < 14%).

**Figure 6:**
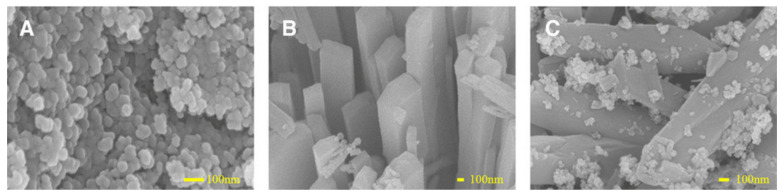
SEM micrographs of Fe_3_O_4_-NH_2_ NPs (**A**), bio-MOF-1 (**B**), and Fe_3_O_4_-NH_2_/bio-MOF-1 (**C**). Reprinted from [75] with permission from Wiley-VCH. Copyright 2018.

In another example, magnetic cobalt adeninate MOFs were synthesized by Zhang and co-workers [75] for the extraction of benzodiazepines from urine and wastewater. The authors anchored Fe_3_O_4_ NPs onto the external surface of cobalt adeninate MOFs by using amino-silane as a linkage (see Figure 6). As can be seen, the magnetic NPs were loaded onto the external surface of bio-MOF well, and the morphology of the framework (bio-MOF-1) was still retained. The biocomposite was applied as an extraction phase in magnetic-assisted dispersive SPE to capture the target compounds, followed by HPLC-MS determination. The analytical performance of the method was evaluated by establishing the recoveries (80–95%), enrichment factors (between 37 and 102), sorbent reusability (at least 10 times without significant losses), and LODs (0.2–2 ng L^−1^). The results revealed that this method exhibited lower LODs than those obtained by the other extraction methods, such as liquid–liquid extraction, SPE, and SPME, among others. The good extraction performance was closely related to the high adsorption affinity, which was mainly due to the hydrogen bonding, the electrostatic and π-stacking interactions between the analytes, and the aromatic acid moieties and Ade present in the bio-MOF.

**Figure 7:**
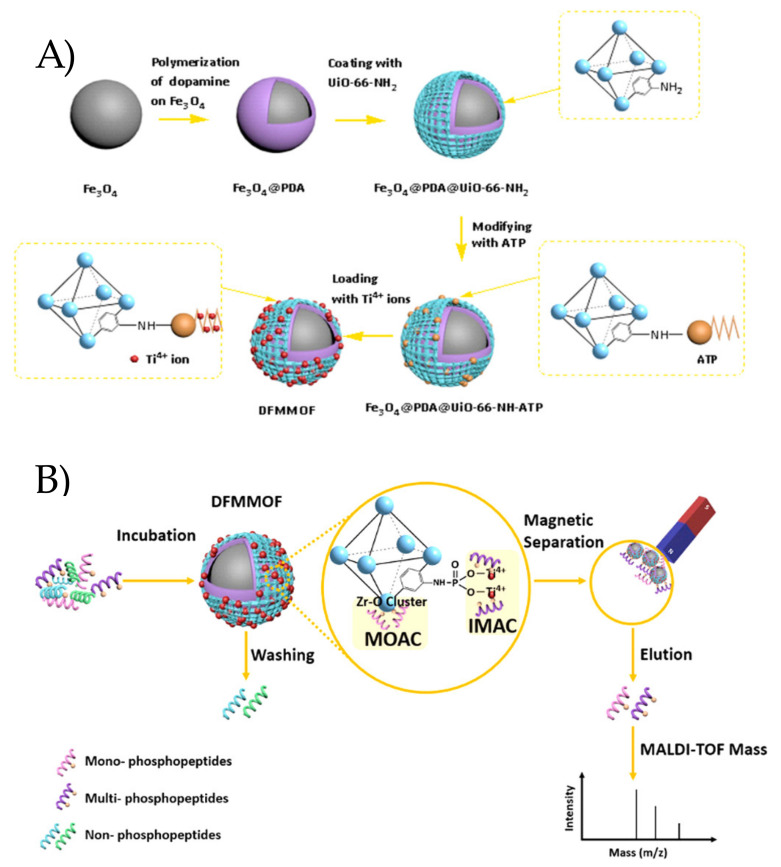
(**A**) Synthesis procedure of biocomposite. (**B**) Schematic representation of Zr-MOF@ATP@Ti for selective capture of phosphorylated peptides. Reprinted with permission from [77]. Copyright 2017 American Chemical Society.

Recently, nucleotides such as adenosine triphosphate (ATP) have been also incorporated in MOFs, taking advantage of its phosphate functionalities for immobilizing metal ions. Thus, Zhou et al. [77] have synthesized a dual-functionalized magnetic zirconium-based MOF for the selective extraction of phosphopeptides. The biocomposite was prepared by modifying UiO-66-NH_2_ MOF onto PDA-coated magnetic spheres and subsequently the immobilization of titanium (IV) ions onto the surface of the obtained ATP-grafted magnetic MOF (see synthetic route in Figure 7A). The material was successfully applied for the capture of phosphopeptides by magnetic dispersive micro-SPE, as shown in Figure 7B. The inherent Zr–O clusters of MOF played the role of metal oxide affinity chromatography (MOAC) sites, whereas the immobilized titanium (IV) ions on the surface of MOF acted as the immobilized metal ion affinity chromatography (IMAC) phase, which provided the resulting material a high metal affinity toward both mono- or multi-phosphorylated peptides. The developed sorbent exhibited a rapid magnetic separation (within 5 s), large surface area (237.9 m^2^ g^−1^), high binding capacity (100 mg g^−1^), and good post-enrichment recovery (84.8%). In addition, the method gave a low detection sensitivity (5 fmol) and high selectivity (β-casein/BSA with a molar ratio of 1:1000) for phosphopeptide enrichment. The bio-MOF was also successfully tested for the identification of phosphopeptides in real samples (human serum and nonfat milk), thus confirming its potential as a promising candidate in the study of phosphoproteome.

Up to now, most of the reported MOFs are constructed from rigid or semi-rigid aromatic carboxylate ligands derived from petrochemical sources; however, the use of other flexible and eco-friendly carboxylate ligands as building blocks or as modulators during MOF synthesis is scarce [78,79,80]. Besides, these flexible frameworks can easily adjust their configurations to adapt to the analyte molecules. As an example, Huang and co-workers [78] used a fatty acid, 8-aminocaprylic acid, as a modulator to prepare the 8-aminocaprylic acid-doped UIO-66 and introduce amino groups into UIO-66. This modulator induced structural defects and additional pore space, which increased the specific surface area and pore volume. The as-synthesized material was coated on the surface of the stainless steel wire with the help of a neutral silicone sealant to be used as SPME fiber. The prepared fibers were used in a head-SPME method combined with GC-MS to evaluate the migration of nitrosamines from latex gloves to saliva. The method showed the following analytical parameters: relative recoveries with average values of 99.1%, LODs of 2.61–6.12 ng L^−1^, and an acceptable fiber-to-fiber reproducibility (RSD values < 11.8%).

Additionally, MOFs have been incorporated into polymers to fabricate certain hybridizations with particular architectures and properties. Among the various polymers, chitosan (CS) stands out, since it acts not only as a continuous phase or support but also as an adsorption adjuvant in hybridizations. The resulting biocomposites would have the merits of high porosity, excellent adsorption ability, and reusability, which are very beneficial for the enrichment of trace targets in aqueous samples [81,82,83]. Thus, Li et al. [81] developed a variety of highly porous MOF/CS foams as sorbents for the extraction of parabens in water samples. Among the prepared composites, MIL-53(Al)/CS foam was selected as the best sorbent candidate to develop an ultrasound-assisted SPE method for the target analytes, followed by UPLC-MS/MS determination. Under the optimal conditions, the method showed the following analytical parameters: recoveries of 78.8–102.1%, LODs of 0.09–0.45 μg L^−1^, and a satisfactory reusability (10 re-uses).

## 3. Conclusions

In this review, recent advances in the synthesis and application of MOF-based affinity sorbents in sample treatment have been presented. MOFs are materials that have a high surface area, tunable porosity, adjustable surface chemistry, and availability of a variety of metals and ligands. However, the combination of MOFs with biomolecules (as ligands) to produce bio-MOFs with enhanced properties (high biocompatibility, low toxicity, multiple coordination sites, hydrophobic/hydrophilic tendencies, chirality, self-assembly properties, specific molecular recognition) is strongly desirable and needs to be investigated. Thus, the availability and variety of biomolecules with desired characteristics can lead to the the development of novel efficient hybrid sorbents with an outstanding selectivity to facilitate the extraction and enrichment process of analytes in complex samples. For example, amino or peptide@MOF composites have demonstrated their potential as enantioselective materials for the adsorption/desorption of analytes through a rationale design in the size and shape of the bio-MOF pore to impart unique host–guest interactions. Additionally, the construction of bio-MOF materials using highly selective bioligands (such as antibodies, aptamers, or MIPs) has demonstrated impressive results. The current bio-MOF developments shown here have been applied to the selective extraction and preconcentration of both small solutes as well as large biomacromolecules in samples with different matrices (e.g., environmental, food, and biological samples). In the coming years, it is expected that the research and construction of bio-MOFs with affinity properties will surely grow due to the continuous advances in material science and other disciplines. Thus, the fabrication of biocomposite sorbents with an enhanced selectivity, extraction capacity, reproducibility, stability, and adaptability for a wide range of applications is foreseen. Additionally, the discovery of novel bioMOFs would undoubtedly expand the scope in other areas, and could answer questions such as their stability and use in biology and the cost-efficiency of mimetic-based MOFs, among other things.

## Figures and Tables

**Table 1:** Applications of bio-MOFs prepared using amino acids as affinity ligands with sample preparation purposes.

Amino Acid Ligand	Analytes	Sample	Method	Remarkable Features	Ref.
Alanine, serine and valine	Carvone (terpenoid)	Ethanol	s-SPE-CD	E.E. (18.6–43.2%)	[23]
Histidine	racemic 1-phenylethanol	Gas	GC	E.E. (76% with R form); good reproducibility	[24]
Methionine	Au(I) and Au(III)	Water	d-SPE-ICP-AES and SEM	R. (~100%);%); Q./T.A. (300/3 h, 600/1 h mg g^−1^) ^a^	[25]
Methionine	Hg(II)	Tap water	d-SPE-ICP-MS	R. (99.95%); Q. (284 mg g^−1^); T.A. <15 min	[26]
Cysteine	N-Linked Glycopeptides	HeLa cell lysate	d-SPE-MALDI-TOF MS	R. (~80%); Q. (150 mg g^−1^); LOD (1 fmol); A.T. (5 min); selectivity (1:50)	[27]
Arginine	Phosphopeptides	Tryptic digest of rat brain lysate	m-SPE-MALDI-TOF MS	LOD (10 fmol); Selectivity (1:1000)	[28]
Serine	B-vitamins	Juices and E.D.	SPE-HPLC-UV	25 mg of sorbent; 5 reuses; Q. (50 mg g^−1^); LOD (0.4–1.12 μg L^−1^); R. (75–123%)	[29]
Cysteine	Glycopeptides and phosphopeptides	H.C.L.P.	MSPE-MALDI-TOF MS	LOD (0.1 fmol mL^−1^); 5 reuses; high selectivity (1:100)	[30]
Cysteine	Cd(II)	Wastewater	m-SPE-ICP-AES UAE-FLD	R.E. (~100); 5 reuses; 50 mg of sorbent; A.T. (10 min); Q. (248.24 mg g^−1^)	[31]
Glycine	Drugs	Water	d-SPE-HPLC (TGA and powder XRD patterns, before and after)	Q. (30–180 mg g^−1^)	[32]
Cysteine	Patulin (toxin)	Apple juice	s-SPE-HPLC-UV	50 mg of sorbent, Q. (4.38 mg g^−1^) A.T. (3 h), non-toxic at <10 mg L^−1^	[33]

s-, d-, m-SPE static, dispersive, magnetic SPE; ICP: inductively coupled plasma; MS: mass spectrometry; AES: atomic emission spectroscopy; SEM: scanning electron microscopy; MALDI-TOF: matrix-assisted laser desorption/ionization time of flight; UAE-FLD: ultrasonic-assisted extraction-fluorescence detection; CD: circular dichroism; TGA: thermogravimetric assay; XRD: X-Ray diffraction; E.D.: energy drinks; H.C.L.P: human crystalline lens proteins; R.: recovery; Q.: adsorption capacity; T.A.: time of analysis; A.T.: adsorption time; R.E.: removal efficiency; E.E.: enantiomeric excess. ^a^ for Au(I) and Au(III), respectively.

**Table 2:** Applications of immune- and antibody-mimic affinity ligands using MOF supports for sample treatment.

Affinity Based MOF Sorbent	Analytes	Matrix	LOD	Remarkable Features	Reference
Ab@ZIF-8/Fe_3_O_4_	*Staphylococcus Aureus*	Milk	300 cfu mL^−1^	Selective in the presence of other bacteria	[59]
Co_3_Fe-MMOF@PDA@Ab	*Aeromonas Hydrophila*	Water	17 cfu mL^−1^	R. (60–70%), Selective	[60]
MOF-5-Apt	PCBs	Fish	0.003–0.004 ng mL^−1^	R. (89–97%); E.F. (1930–2304); RSD. (<5%)	[62]
Fe_3_O_4_@PDA@UiO-66-Apt	PCBs	Soil	0.010–0.015 ng mL^−1^	R. (89–95%); Q. (195–218 ng mg^−1^); 60 reuses	[63]
Cu/UiO-66@Apt	PCBs	Serum	2.1 pg mL^−1^	R. (88–102%); Q. (37.17 μg g^−1^); 10 reuses; A.T. (30 min); RSD. (<4%); Selective	[64]
Apt-MMIL-101	OTA	Corn and peanut	0.067 ng L^−1^	R. (83–108%); Q. (553 pmol mg^−1^); 12 reuses; E.F. (167); RSD (12%); A.T. (40 min); D.T. (30 min)	[65]
Cu/UiO-66@Apt	CAP	Fish	0.09 nmol L^−1^	R. (97–104%); A.T. (40 min); Selective	[66]
MOF-177@MIPs	S-amlodipine	–	–	Q. (536 mg g^−1^); I.F. (5.79)	[67]
NH_2_-MIL-101@MIP	DS	Urine	–	R. (88–102%); Q. (15.78 mg g^−1^); RSD. (7%); A.T. (20 min); Selective	[68]
UiO-66-NH_2_@MIP	AT (B1, B2, G1, G2)	Grain	90–130 ng kg^−1^	R. (74–99%); Q. (4–10 mg g^−1^); RSD. (<6%); Selective	[69]
UiO-66-NH_2_@MIP	TCs	Chicken muscle	0.2–0.6 ng g^−1^	R. (70–95%); Q. (2200–3000 mg g^−1^); RSD. (<12%); A.T. (15 min); E.F. (18–37)	[70]
MIL-101@MIP	Metolcarb	Pear juice	0.0689 mg L^−1^	R. (74–96%); Q. (1.32 mg g^−1^); Selective; RSD. (<4.2%)	[71]

Ab: antibody; MMOF: magnetic MOF; PDA: polydopamine; Apt: aptamer; MIP: molecular-imprinted polymer; PCBs: polychlorinated biphenyls; OTA: ochratoxin A; CAP: chloramphenicol; DS: diclofenac sodium; AT: aflatoxins; TCs: tetracyclines; cfu: colony-forming unit; R.: recovery; E.F.: enrichment factor; RSD: relative standard deviation; Q.: adsorption capacity; A.T.: adsorption time; D.T.: desorption time; I.F. imprinted factor.

**Table 3:** Applications of other affinity ligands using MOF supports for sample treatment.

Biomolecule Ligand	Method	Analytes	Sample	R. (%)/LOD	Remarkable Features	Ref.
Adenine	d-SPE-UV	PCPs	Water	–/–	A.T. (12 h); 3 mg sorbent; 4 reuses	[73]
Adenine	SPME-GC-FID	PAHs and OCPs	Water	80–111/2.2–2.3 ng L^−1^	A.T. (40 min); D. T. (300 s)	[74]
Adenine	m-SPE-LC-MS	Benzodiazepines	Urine and water	80–95/0.7–2.4 ng L^−1^	A.T. (40 min); D.T. (15 min); E.F. (37–102); 10 reuses	[75]
Adenine	SPME-GC-FID	PAHs	Water	80–115/20–5600 ng L^−1^	A.T. (30 min) D.T. (5 min)	[76]
ATP	d-SPE-MALDI-TOF	Phosphopeptides	Serum and milk	85/5 fmol	Q. (100 mg g^−1^); 3 reuses; A.T. (30 min) D.T. (15 min) Low RSD; Selective (1:1000)	[77]
8-aminocaprylic acid	SPME-GC-MS	Nitrosamines	Latex globes	85–113/2.6–6.1 ng L^−1^	A.T: (30 min); RSD (<12%)	[78]
Lactate	d-SPE-NCI-GC-MS	PBDEs	Environmental water	84–102/0.08–0.15 ng L^−1^	RSD (<12%)	[79]
Citric anhydride	d-SPE-AAS	Pb(II)	Water	80/–	Q. (390 mg g^−1^); A.T. (120 min)	[80]
Chitosan	d-SPE-UPLC-MS/MS	Parabens	Water	79–102/90–450 ng L^−1^	A.T. (20 min); D.T. (15 min); 10 reuses; RSD (<7.4%)	[81]
Sodium Alginate/Chitosan	d-SPE-HPLC-UV	Bisphenol A	Wastewater	–/–	Q. (101–137 mg g^−1^); A.T. (18 h)	[82]
Chitosan	d-SPE-UV	Iodine	Wastewater	96/–	Q. (400 mg g^−1^); A.T. (90 min); 5 reuses	[83]
Histamine	d-SPE-GC-FID	OPPs	Water and juice	92–100/30–210 ng L^−1^	E.F. (803–914); 8 reuses	[84]
Urea	SPE MALDI-TOF-MS	Phosphopeptides	Nonfat milk	–/10^−10^M	Selectivity (1:200); 5 reuses	[85]

ATP: adenosine triphosphate; d-SPE-UV: dispersive solid-phase extraction ultraviolet detection; SPME; micro-SPE; GC-FID: gas chromatography flame ionization detector; m-SPE: magnetic SPE; HPLC (LC)-MS: high-performance liquid chromatography mass spectrometry detection; NCI: negative chemical ionization; AAS: atomic absorption spectrometry; MALDI-TOF: matrix-assisted laser desorption/ionization; TOF: time of flight; UPLC: ultra-HPLC; FID: flame ionization detector; PCPs: personal care products; PAHs: polycyclic aromatic hydrocarbons; OCPs: organochlorine pesticides; PBDEs: polybrominated diphenyl esthers; OPPs: organophosphorus pesticides; Q.:adsorption capacity; A.T.: adsorption time; RSD: relative standard deviation; D.T.: desorption time; E.F.: enrichment factor.

## Abbreviations

MOF	Metal-organic framework
Bio-MOFs	Biological metal-organic frameworks
MS	Mass detector
SPE	Solid-phase extraction
MIPs	Molecular imprinted polymers
NPs	Nanoparticles
PDA	Polydopamine
LOD	Limit of detection
MALDI	Matrix-assisted laser desorption/ionization
TOF	Time of flight
HPLC	High-performance chromatography
RSD	Relative standard deviation
GSH	Glutathione
Ee	Enantiomeric excess
FLD	Fluorescence detection
ZIF	Zeolitic imidazolate framework
CS	Chitosan
CD	cyclodextrin
PCBs	Polychlorinated biphenyls
Ade	Adenine
SPME	Solid-phase micro-extraction
PAHs	Polycyclic aromatic hydrocarbons
FID	Flame ionization detector
